# Comparison between orogastric tube/bougie and a suction calibration system for effects on operative duration, staple-line corkscrewing, and esophageal perforation during laparoscopic sleeve gastrectomy

**DOI:** 10.1007/s00464-015-4399-z

**Published:** 2015-07-14

**Authors:** Michel Gagner, Rose Y. Huang

**Affiliations:** Department of Surgery, Hospital du Sacre Coeur, Montreal, QC Canada; Boehringer Laboratories, LLC, Phoenixville, PA USA

**Keywords:** Sleeve gastrectomy, Calibration system, Bougie, ViSiGi 3D™, Corkscrewing, Delineation

## Abstract

**Introduction:**

Placement of a bougie for sleeve sizing during laparoscopic sleeve gastrectomy (LSG) is recommended. We compared this standard with a suction calibration system (SCS) that performs all functions with one insertion, and measured each step’s duration.

**Methods:**

Primary LSG was performed using a bougie and SCS in alternating order. Number of tube movements to achieve optimal placement, durations of decompression, leak testing, and overall operative time, and remnant linear measurements were obtained.

**Results:**

LSG was performed in 26 patients (15 women, 11 men; mean age 36.8 years; mean BMI 45.3 kg/m^2^). The mean number of tube movements was significantly greater for the bougie than for the SCS (8.13 vs. 3.58; *p* < 0.0001). Percent reductions achieved using the SCS were: time to full decompression of the stomach, 62 % (21 vs. 8 s; *p* < 0.138); tube placement, 51 % (101 vs. 49 s; *p* < 0.0001); leak testing, 78 % (119 vs. 26 s; *p* < 0.0003); and mean operative duration (from tube insertion to end of stapling), 21 % (875 vs. 697 s; *p* < 0.019). Variance of the staple-line distance, measured from the greater curvature to the staple line, was 1.64 and 0.92 for the bougie and SCS, respectively, indicating a reduction in corkscrewing, for a 43.9 % straighter sleeve.

**Conclusion:**

SCS maintained the gastric wall in place, thereby preventing corkscrewing, and reducing total operating time. Reducing the number of tube insertions may prevent esophageal damage and accidental tube stapling.

A critical step in the internationally recognized laparoscopic sleeve gastrectomy (LGS) is ensuring sleeve-size consistency. According to the International Sleeve Gastrectomy Expert Panel Consensus Statement [1], 100 % of the surgeons surveyed the sleeve size with a bougie. However, clinical disadvantages of using a bougie include accidental stapling across the tip, esophageal perforation, and staple-line corkscrewing.

With super-obese patients, visualization of a bougie or an intraluminal tube placement is often difficult (e.g., orogastric [OG] tube and temperature probe). Most surgeons rely on tactile feedback or ask the anesthesiologist to move the bougie to confirm placement. This difficulty in locating and securing intraluminal tubes inside bariatric patients often leads to accidental stapling across these tubes. A multicenter study reported the rate of this complication to be 1 in 132 [2]. Such accidents could result in conversion of LSG to gastric bypass, or even leakage. Also, the tungsten-filled and gravity-driven bougie has a tendency to spring away from the lesser curvature with the patient in reverse Trendelenburg position. Often the anesthesiologist has to hold the bougie in place throughout the stapling of the stomach.

LSG also requires the insertion of an OG tube, for stomach decompression before sizing and leak testing after sizing. Multiple tubes traversing the esophagus of a patient in reverse Trendelenburg position amplify the risk of esophageal perforation, which can result in treatment costs of over $100,000 [3].

Surgeons are instructed to keep LSG staple lines within the same frontal plane to avoid sleeve corkscrewing and uneven anterior and posterior traction on the staple line, both of which can result in a functional obstruction not easily remedied endoscopically [4]. Before each staple firing, surgeons often flip the stomach back and forth to check for excess tissue on the posterior side. Sliding tissues around the bougie can cause inconsistent tension on the anterior or posterior side of the stomach during and after stapling.

The patient safety profile can be improved by reducing the number of steps and clinical risks with LSG. The aim of this study was to compare aspects of the procedure, including visual confirmation of device position, intraoperative duration of all steps, staple line, and number of device movements to achieve optimal placement, between current practice and a new calibration system, the ViSiGi 3D™ (Boehringer Labs, LLC, Phoenixville, PA, USA), which employs a safe level of suction and performs all functions with one insertion.

## Materials and methods

This was a single-center, single-surgeon prospective study. Informed consent was obtained from all individual participants included in the study. Primary LSG was performed using either a 40-Fr bougie or a 40-Fr suction calibration system (SCS), the ViSiGi 3D™, in alternating order, with the decision to use either device in the first case being determined at random. Sex, age, body mass index (BMI), and comorbidity information were recorded for each patient.

Total operating time was defined as the time spent to complete all steps involving use of an intraluminal tube (i.e., bougie, SCS, and OG tube). These steps included: stomach decompression, device positioning, stapling, and leak testing. The criteria to record each of these steps are detailed in Table 1. Duration of stomach dissection, hernia repair, and oversewing were excluded.

**Table 1:** Time measurement criteria for each step involving an intraluminal tube

Measurement	Start	End
Stomach decompression	When anesthesiologist was inserting the OG tube or SCS. In the case of anesthesiologist already placed OG tube before insufflation, the decompression time starts if the surgeon needs additional time/measure to ensure complete decompression	When the surgeon noted that the stomach was fully decompressed
Positioning time	When the anesthesiologist was instructed to advance the SCS, or to remove the OG tube and insert the bougie	When the surgeon was ready to fire the first staple load
Stapling time	When the stapler was inserted	When last staple load was fired
Leak testing	When the anesthesiologist was instructed to remove the bougie and insert the OG tube, or to inject methylene blue into the SCS	When the anesthesiologist was instructed to remove all intraluminal tubes

To determine the staple-line straightness, three measurements were taken at three predetermined locations on the inflated, excised gastric specimen: circumference, and distances from the greater curvature to the staple line anteriorly and posteriorly (Figs. 2, 3). The three predetermined locations were the midpoint of the staple line and the points 5 cm above and below the midpoint. The variance of deviation from the midline at these locations was calculated for each specimen. To standardize the amount of inflation, each specimen was connected to the same gas source for 5 s through an insufflation needle.

The number of device movements was recorded in both groups. This included the number of intraluminal devices inserted, as well as the number of times the surgeon inquired about the status/location of the devices; e.g., asking the anesthesiologist “Is it in?” or “Is it out?” or to move or hold the device in place, or to insert or remove any intraluminal tube, and any repositioning of the device.

**Fig. 1:**
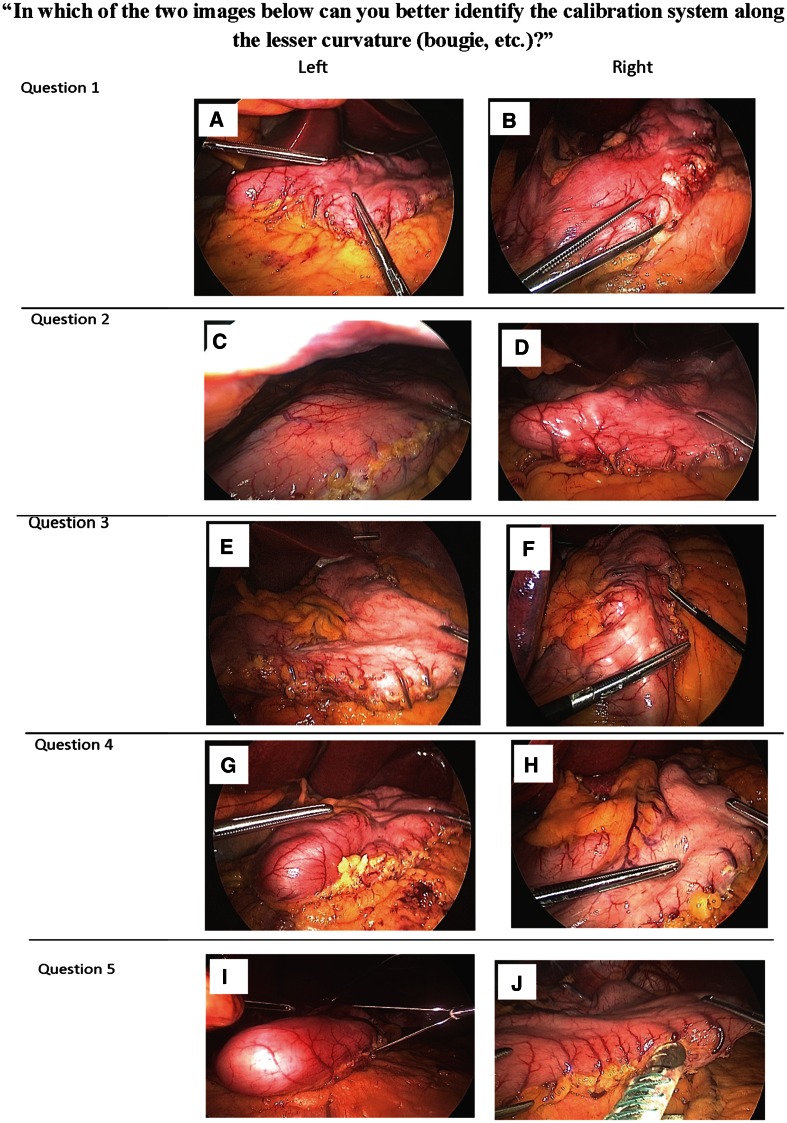
Survey questions. (**A**) ViSiGi case #2, (**B**) bougie case #1, (**C**) bougie case #23, (**D**) ViSiGi case #4, (**E**) bougie case #12, (**F**) ViSiGi case #9, (**G**) ViSiGi case #13, (**H**) bougie case #6, (**I**) ViSiGi case #19, (**J**) bougie case #14

Visual confirmation of device position was captured in an intraoperative photograph of each stomach prior to the first staple firing. To assess the degree of visualization of both devices, five out of thirteen photographs from each study group were randomly chosen and paired side by side for comparison (Fig. 1). During the 19th World Congress IFSO 2014 (International Federation for the Surgery of Obesity and Metabolic Disorders) in Montreal, Canada, 273 attending surgeons were asked to participate in a survey in which they answered the same question for all five pairs of photographs: “In which of the two images below can you better identify the calibration system along the lesser curvature (bougie, etc.)?” The result of the survey demonstrates whether there is a visual difference between bougie and SCS, which directly relates to the safety and effectiveness of the device.

### Statistical analysis

Statistical analysis was performed with a two-sample *t* test assuming unequal variance. In addition, staple-line straightness analysis was performed with one-way analysis of variance (ANOVA) using StatPlus^®^ software (AnalystSoft, Inc., Alexandria, VA, USA).

**Fig. 2:**
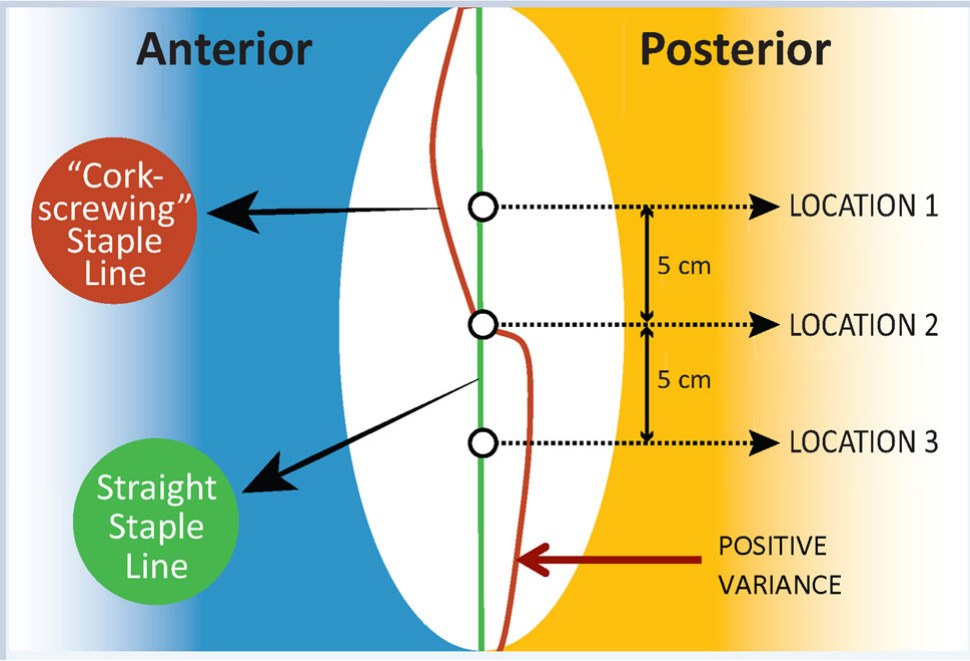
Diagram depicting measurement to determine straightness of staple line. Measurements of the circumference, anterior, and posterior distance were taken at three locations along the inflated excised gastric specimen: location 1: 5 cm above the midpoint of the staple line, location 2: midpoint of the staple line, location 3: 5 cm below the midpoint of the staple line

**Fig. 3:**
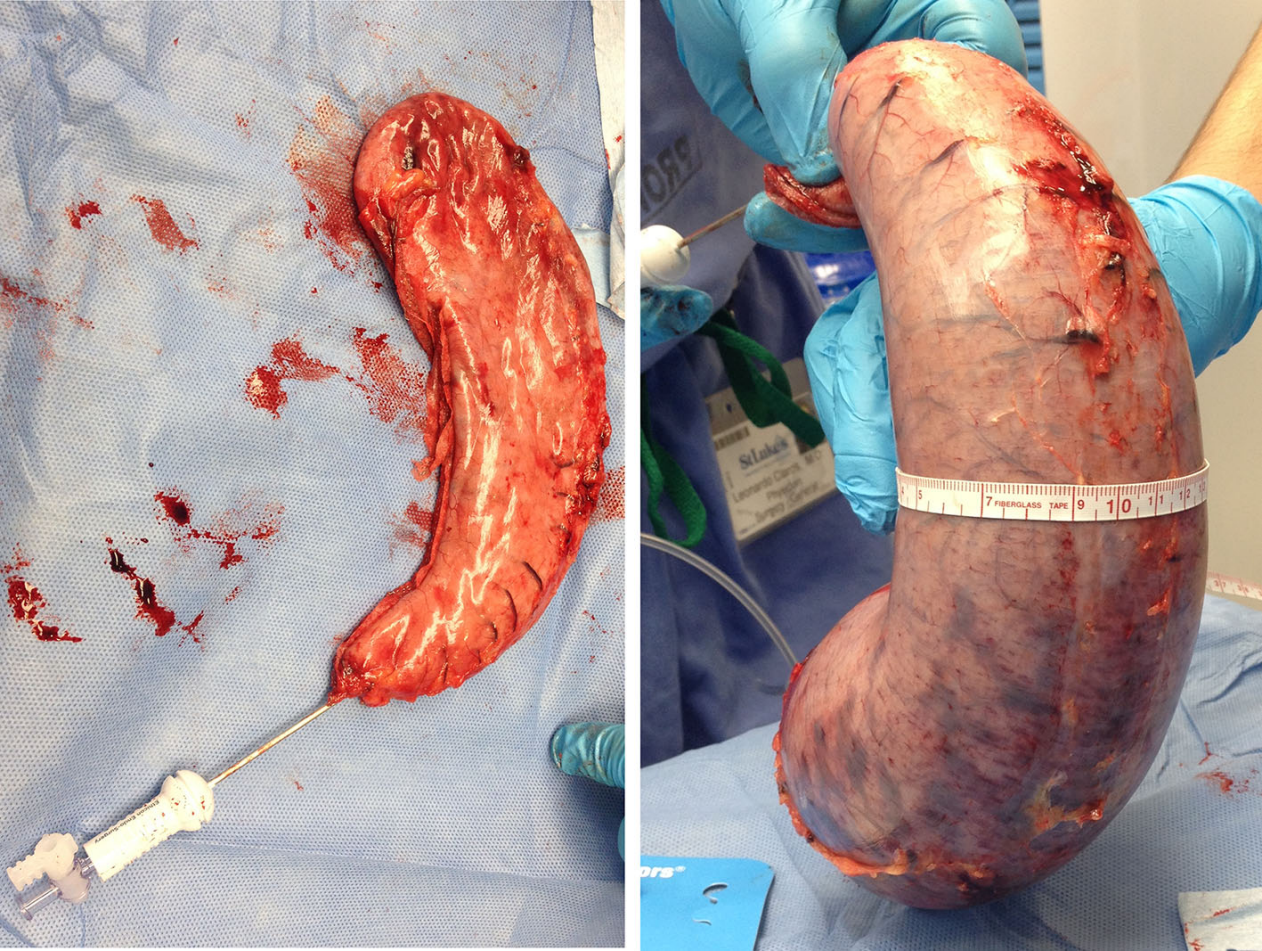
Measuring the straightness of the staple line

The equation is given as follows:

$$ V_{{\text{[top}}, \,{\text{middle}},\, {\text{or}} \,{\text{bottom}}]} = D_{\text{anterior}} - D_{\text{posterior}},$$

where *D*_anterior_ represents anterior distance from greater curvature to staple line, *D*_posterior_ represents posterior distance from greater curvature to staple line and *V*_[top, middle, or bottom]_ represents the difference between the two at one of the three points along the greater curvature. A positive number indicates a posteriorly deviated staple line, and a negative number indicates an anteriorly deviated staple line (Fig. 2). For a straight staple line, the difference between the distances from greater curvature to anterior and posterior edges would be the same at all three predetermined locations (Fig. 3),

$$ V_{\text{top}} = \, V_{\text{middle}} = \, V_{\text{bottom}}. $$

**Fig. 4:**
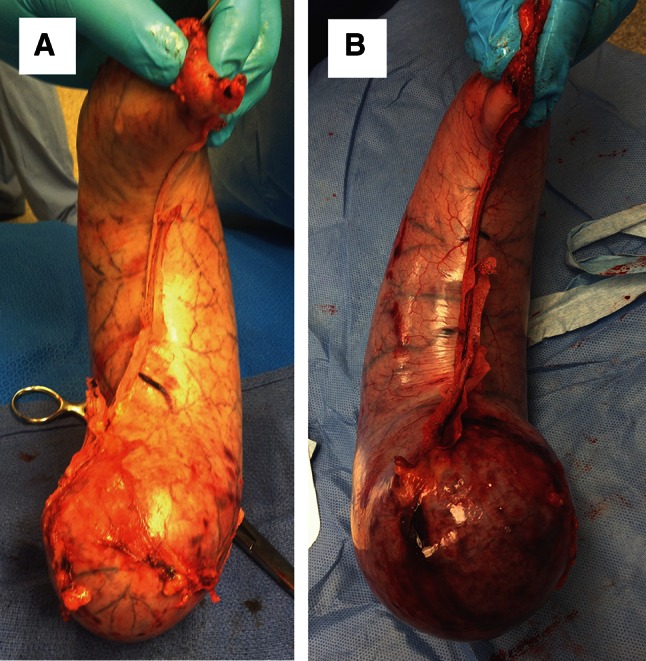
Staple lines. (**A**) Corkscrew and (**B**) straight staple lines

A variance of zero between *V*_top_, *V*_middle_, and *V*_bottom_ indicates identical values signifying a straight staple line. A small variance indicates closer data points, whereas a large variance indicates more widespread data points representing a greater degree of corkscrewing (Fig. 4).

### New technology

**Fig. 5:**
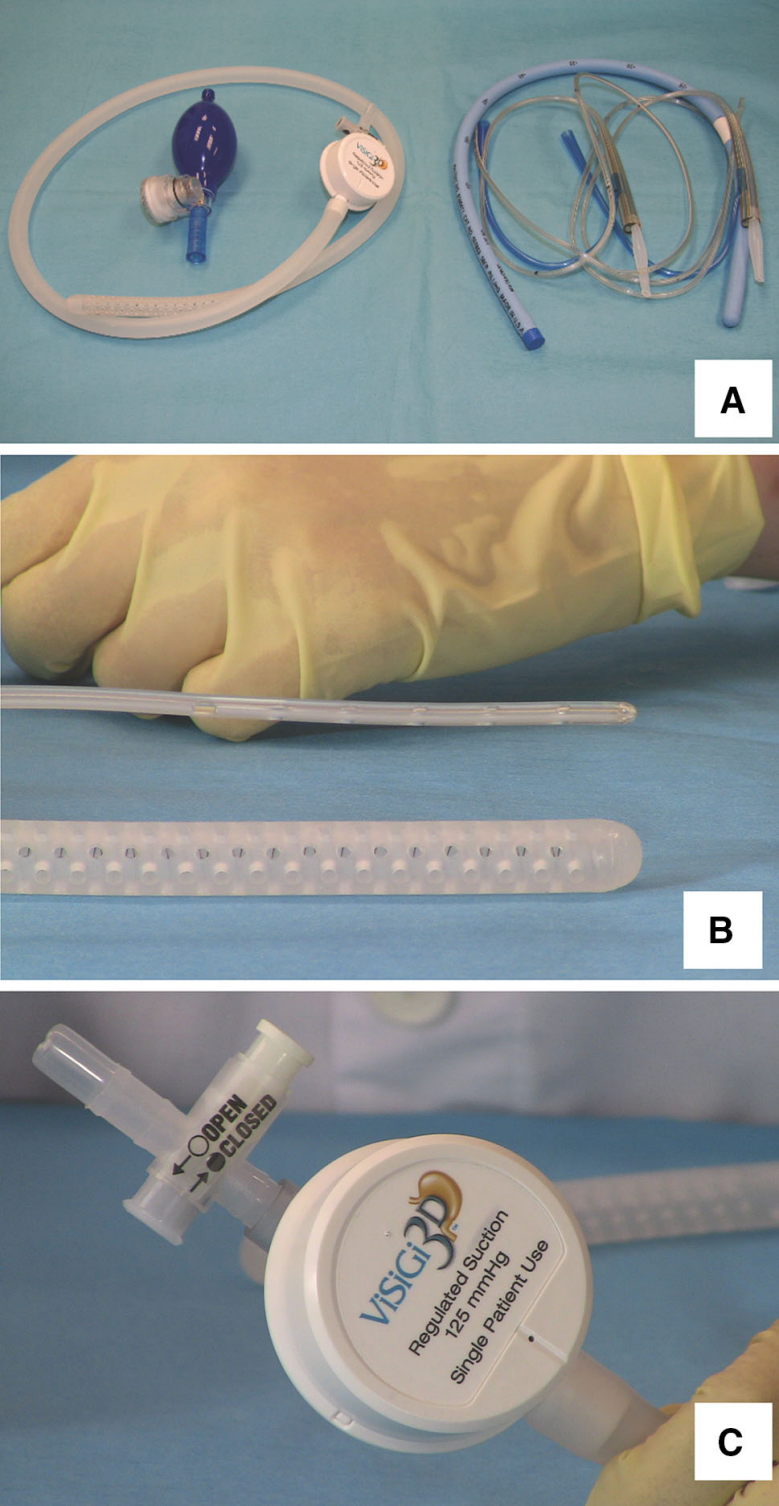
(**A**) One-step ViSiGi 3D™ versus multi-step orogastric tube/bougie system. (**B**) Circumferential fenestration pattern enabling flexibility of the ViSiGi 3D™. (**C**) Integral regulator safely decompresses the stomach at 125 mmHg regulated suction

The ViSiGi 3D™ calibration system is indicated for use in gastric and bariatric surgical procedures for the application of suction, stomach decompression, drainage of gastric fluids, and irrigation, and to serve as a sizing guide. Compared with a bougie, which must be used in combination with other apparatuses, the anesthesiologist inserts the ViSiGi 3D™ only once at the beginning of the case and removes it after stapling and leak testing are completed (Fig. 5a). The circumferential fenestration pattern at the distal end of the ViSiGi 3D™ facilitates complete suction decompression of the stomach prior to trocar placement (Fig. 5b). This pattern also applies uniform suction on both the anterior and posterior sides of the stomach, creating equal tension on the staple line. The integrated suction regulator reduces the high pressure normally found in the operating room (600 mmHg or more) to 125 mmHg, a clinically safe level for gastric tissues (Fig. 5c). The combination of the one-way valve and the fenestration allows air or methylene blue to be passed, so leak testing can be performed with the device in place. The ViSiGi 3D™ also includes an accessory bulb that delivers controlled air pressure during a leak test.

## Results

Enrollment of 26 patients (15 females, 11 males; mean age 36.8 years [range 14–74 years]) was completed in 2014. Mean BMI was 45.3 kg/m^2^ (range 35.0–61.0 kg/m^2^, Table 2). The randomized, blinded survey was completed at IFSO 2014 by 273 surgeons, who were not informed about the two different methods prior to taking the survey but were instructed simply to select the picture in which the calibration system could be better visualized. Upon visual inspection of the images, 91.5 % of the participating surgeons stated they were better able to identify the calibration system along the lesser curvature in pictures involving the ViSiGi 3D™ (Table 3).

**Table 2:** Patient demographics

	Female (*N* = 15)	Male (*N* = 11)
Min BMI	39.0	35.0
Max BMI	61.0	60.6
Mean BMI ± SD	45.7 ± 6.7	44.7 ± 8.2
Min age	22	14
Max age	61	74
Mean age	36 ± 10.8	38 ± 17.0

*BMI* body mass index (kg/m^2^), *max* maximum, *min* minimum, *SD* standard deviation

**Table 3:** Survey responses

“In which of the two images below can you better identify the calibration system along the lesser curvature (bougie, etc.)?”		
	Left	Right
Question 1	96.3 % (ViSiGi)	3.7 % (Bougie)
Question 2	1.1 % (Bougie)	98.9 % (ViSiGi)
Question 3	7.0 % (Bougie)	93.0 % (ViSiGi)
Question 4	81.6 % (ViSiGi)	18.4 % (Bougie)
Question 5	87.5 % (ViSiGi)	12.5 % (Bougie)

Table 4 presents a comparison of overall operating time as well as the length of each recorded interval between the ViSiGi 3D™ and the OG -bougie-OG tube system. Total operating time involving the use of an intraluminal tube was significantly shorter with the ViSiGi 3D™ than with the OG-bougie-OG system (*p* < 0.019). Mean time to manipulate and place an intraluminal tube along the lesser curvature was 51 % shorter with the ViSiGi 3D™ (*p* < 0.0001). Mean time to complete the leak test was significantly shorter with the ViSiGi 3D™ than with the bougie (*p* < 0.003).

**Table 4:** Mean time to complete each step of sleeve gastrectomy with an intraluminal tube

Mean time (sec)	Bougie	SCS^a^	% Save	*p* value
Fully decompressed stomach	21.0	8.0	61.9	0.138
Tube placement	101.0	48.6	51.9	0.000
Each staple firing	137.6	119.5	13.2	0.085
Leak test	119.4	26.5	77.8	0.000
Total operative time (excluding complications, hernia, oversew, etc.)	875.3	697.2	20.3	0.019

*SCS* suction calibration system; *sec* seconds

Ten specimens were analyzed for straightness of the staple line. During the removal process, some specimens were punctured and not able to sustain pressure. When measuring the anterior and posterior distances from greater curvature to staple line, the average variance of the staple-line deviation was 1.64 (range 0.25–3.08) for the bougie and 0.92 (range 0.08–2.86) for the ViSiGi 3D™, or a 43.9 % straighter staple line (Table 5; Fig. 4).

**Table 5:** Summary of staple line straightness measurements

Location 1—top			Location 2—middle			Location 3—bottom			Mean variance ± SD
*D*_anterior_	*D*_posterior_	*V*_top_	*D*_anterior_	*D*_posterior_	*V*_middle_	*D*_anterior_	*D*_posterior_	*V*_tottom_	
*Bougie* (*cm*)									
12.0	10.5	1.5	8.5	9.5	−1.0	6.0	4.0	2.0	2.85 ± 0.83
13.5	12.0	1.5	9.0	8.5	0.5	6.0	5.0	1.0	0.25 ± 0.5
8.0	9.0	0.1	11.0	10.0	1.0	13.0	10.5	2.5	3.08 ± 1.76
14.5	12.5	2.0	11.0	9.0	2.0	9.5	9.5	0.0	1.33 ± 1.15
12.0	12.0	0.0	8.5	10.5	−2.0	7.0	8.0	−1.0	1.00 ± 1.0
12.0	11.0	1.0	9.0	9.0	0.0	7.5	9.0	−1.5	1.58 ± 1.26
*SCS* (*cm*)									
12.0	10.8	1.2	11.2	9.0	2.2	7.7	8.8	−1.1	2.86 ± 1.69
10.0	12.5	−2.5	5.0	10.0	−2.0	5.0	6.5	−1.5	0.25 ± 0.5
10.8	10.2	0.6	6.5	8.0	1.5	7.5	5.5	2.0	0.50 ± 0.71
13.0	11.5	1.5	10.0	8.0	2.0	6.0	4.0	2.0	0.08 ± 0.29

*SD* standard deviation, *SCS* suction calibration system

The number of device movements for both groups was recorded. The average number of tube movements was 8.1 (range 8–11) for the OG-bougie-OG system versus 3.6 (range 3–4) for the ViSiGi 3D™ (*p* < 0.0001). Typical bougie movements included introducing the OG tube for stomach decompression, removing the OG tube, inserting the bougie for sleeve sizing, surgeon asking the question: “Is there anything else inside the stomach?” holding the bougie in place during stapling, removing the bougie, inserting the OG tube for leak testing, and removing the OG tube.

## Discussion

According to the most recent International Sleeve Gastrectomy Expert Panel Consensus statement [5], stricture formation should be avoided during sleeve gastrectomy. Additionally, among concepts found to be most important to experts: 75 % felt that “maintaining symmetrical lateral traction while stapling will reduce potential strictures”; 82 % felt the “use of a bougie when stapling the incisura angularis will result in decreased incidence of strictures,” and all experts surveyed agreed that “the incisura angularis is a potential stricture site.”

However, a bougie used for calibration has no internal stability. If gastric tissues are overextended during stapling, the elasticity of the tissues will lead to tissue retraction, and may result in stricture, especially near the incisura angularis. Uniform circumferential suction allows the anterior and posterior walls to envelope the SCS, which guides staple placement, decreasing the incidence of strictures.

With a 1 % incidence, stricture is the second most prevalent early complication, and most symptomatic within 6 weeks postoperatively [6–12]. Treatment options vary, and some authors are proponents of a silicone-covered nitinol stent in an attempt to cause scarred permanent dilatation. One author has advocated several forms of gastroplasty to keep the sleeve intact, such as a stapled end-to-end gastrogastrostomy with transoral anvil passage, local excision with a vagus-preserving, hand-sewn stapled end-to-end gastrogastrostomy, or a transverse linear gastroplasty. However, Roux-en-Y gastric bypass remains the best surgical treatment for persistent strictures after endoscopic treatments have failed.

Corkscrewing of the staple line has been discussed at meetings and in books [4, 15]; but this form of *functional kink* or *spiraling* has not been well studied. Because of gastric tissue elasticity, it is possible to apply uneven tension on the anterior and posterior walls as the posterior stomach is examined for redundant tissue during stapling. Our results suggest that the ViSiGi 3D™ provides controlled and uniform suction, which may enable symmetrical lateral traction, thus preventing corkscrewing. This can potentially decrease the incidence of dysphagia and malnutrition, including water-soluble vitamin deficiencies. Prevention is preferable to excision of a corkscrewed sleeve and conversion to Roux-en-Y gastric bypass.

Perforation, which can occur if bougie or the tube used is too rigid, is a complication to both patients and hospitals [3]. The ViSiGi 3D™ incorporates a fenestration pattern that makes the distal tip more flexible, which may contribute to reducing the risk of perforation. Also, with the OG-bougie-OG system, the anesthesiologist has to insert and remove three intraluminal tubes during the surgery. Thus, consolidation of these three tasks into one using the ViSiGi 3D™ confers an additional threefold reduction in the risk of perforation.

Additionally, intraluminal tubes such as temperature probes, OG tubes, and bougies have been inadvertently transected during stapling and creation of the sleeve [2, 13, 14]. At any time during surgery, a bougie can slip out of the unfinished sleeve, after which it can be easily transected proximally. Accidental stapling of these tubes can cause significant morbidity and require prolonged operating time for repair. In certain cases, postoperative complications may include stricture or leak. A calibration system that is easily visualized and stays anchored along the lesser curvature provides a clinical advantage. In our survey of surgeons, 91.5 % found the ViSiGi 3D™ enabled clearer, more direct visualization of the device and made the stapling process easier and safer.

By integrating multiple steps into one, the ViSiGi 3D™ significantly reduced the operating time needed to complete each step of a sleeve gastrectomy and therefore the total operating time. Based on the clinical benefits found in the present study, we recommend incorporating the ViSiGi 3D™ into the LSG protocol.

Our study objectively examined and compared the degree of staple-line spiraling in LSG. Larger studies are needed to investigate the effect of suction on corkscrewing of the staple line. Despite our small sample size, the preliminary results of the present study appear encouraging. In addition, the limit of this study includes the inability to blind the surgeon to whether the patient was receiving a bougie or a SCS, which could potentially introduce bias. Due to the mechanism of SCS, which requires the surgeon to instruct the anesthesiologist turning on and off suction, the surgeon needs to know which device was using ahead of time. The blinded portion of the study was the survey conducted during IFSO. To ensure objectivity of the interpretation, the 273 surgeons were neither informed about the study nor aware of a new device. They were simply asked the question: “In which of the two images below can you better identify the calibration system along the lesser curvature (bougie, etc.)?”
